# Effect of Popcorn (*Zea mays* var. *everta*) Popping Mode (Microwave, Hot Oil, and Hot Air) on Fumonisins and Deoxynivalenol Contamination Levels

**DOI:** 10.3390/toxins13070486

**Published:** 2021-07-13

**Authors:** Pierre Schambri, Sophie Brunet, Jean-Denis Bailly, Didier Kleiber, Cecile Levasseur-Garcia

**Affiliations:** 1 Nataïs, 32130 Bézéril, France; p.schambri@popcorn.fr (P.S.); s.brunet@popcorn.fr (S.B.); 2 Equipe Biosynthèse et Toxicité des Mycotoxines, ENVT, UMR Toxalim, Université de Toulouse, 31000 Toulouse, France; jean-denis.bailly@envt.fr; 3 Physiologie, Pathologie et Génétique Végétales (PPGV), Toulouse University, INP-Purpan, 31300 Toulouse, France; didier.kleiber@purpan.fr; 4 Laboratoire de Chimie Agro-Industrielle (LCA), Toulouse University, INRAE, INPT, INP-Purpan, 31000 Toulouse, France

**Keywords:** deoxynivalenol, fumonisins, popcorn, popping, mycotoxins reduction

## Abstract

Mycotoxins are secondary metabolites that are produced by molds during their development. According to fungal physiological particularities, mycotoxins can contaminate crops before harvest or during storage. Among toxins that represent a real public health issue, those produced by *Fusarium* genus in cereals before harvest are of great importance since they are the most frequent in European productions. Among them, deoxynivalenol (DON) and fumonisins (FUM) frequently contaminate maize. In recent years, numerous studies have investigated whether food processing techniques can be exploited to reduce the levels of these two mycotoxins, which would allow the identification and quantification of parameters affecting mycotoxin stability. The particularity of the popcorn process is that it associates heat treatment with a particular physical phenomenon (i.e., expansion). Three methods exist to implement the popcorn transformation process: hot air, hot oil, and microwaves, all of which are tested in this study. The results show that all popping modes significantly reduce FUM contents in both Mushroom and Butterfly types of popcorn. The mean initial contamination of 1351 µg/kg was reduced by 91% on average after popping. For DON, the reduction was less important despite a lower initial contamination than for FUM (560 µg/kg). Only the hot oil popping for the Mushroom type significantly reduced the contamination up to 78% compared to unpopped controls. Hot oil popping appears to provide the most important reduction for the two considered mycotoxins for both types of popcorn (−98% and −58% average reduction for FUM and DON, respectively).

## 1. Introduction

Mycotoxins are secondary metabolites synthesized by numerous fungal species. Of special importance are molds of the genus *Fusarium* because they colonize cereals before harvest and may produce some toxins of major importance for food safety such as deoxynivalenol (DON) and fumonisins (FUM). Rodrigues and Naehrer estimated, through a global worldwide study, the prevalence at 72% for DON and 60% for FUM in corn samples from Central Europe [1]. More widely, Gruber-Dorninger, Jenkins, and Schatzmayr, in a 10-year survey on a maize sample panel taken from all continents, showed prevalence rates of 67% and 80% for DON and FUM respectively [2]. DON is a mycotoxin produced by molds of the genus *Fusarium* (*F. graminearum, F. culmorum, F. sporotrichoïdes*,...). It is a common contaminant of cereals especially wheat but also corn. This type B trichothecene has strong emetic properties for animals due to its affinity with dopamine receptors in the brain. Together with its acute and chronic toxicity as well as its worldwide distribution, make DON a significant concern to health authorities [3]. Among the effects on humans, this mycotoxin can cause immunosuppression and digestive toxicities [4]. DON has been associated with some epidemics of gastroenteritis with diarrhea, nausea, abdominal pain, fever, and vomiting in Asia in the last 50 years [5]. FUM are also produced by *Fusarium* species and more especially *F. verticillioïdes* and *F. proliferatum*. These metabolites mostly contaminate corn and the most widespread members of this group of toxins are FUM B1 and B2 (FB1 and FB2) [6]. Studies have revealed the probable role of FB1 (the most toxic compound) in the occurrence of certain cancers (especially esophageal cancers) in several regions of the world as well as its hepatotoxic and nephrotoxic properties [6].

Given that mycotoxins generate various disorders in humans and animals, they are subject to regulations in different countries such as in Europe [7,8]. In particular, these regulations cover toxins produced by *Fusarium* species because they are prevalent in grain and produce the mycotoxins DON and FUM (for maize intended for direct human consumption, the toxin concentrations are limited at 750 µg/kg for DON and 1000 µg/kg for FUM) [7,8].

In general, mycotoxins are stable molecules, able to resist most of the food processes. As an illustration, DON remains stable from 170 to 350 °C, with no reduction in concentration being observed after 30 min at 170 °C [3]. However, data on the heat resistance of DON appear sometimes contradictory [9]. For instance, cooking pasta in boiling water does not affect the levels of DON, whereas other studies claim to the contrary that this mycotoxin is water soluble and can therefore be removed by cooking in boiling water [9,10,11,12,13,14]. Visconti et al. observed reductions of up to 80% in DON levels after cooking spaghetti in boiling water [15]. Finally, Sobrova et al. claimed that frying DON-contaminated food in oil makes no significant reduction in the levels of DON [3]. In the same way, it was reported that oil popping (frying of the grains in oil) does not affect DON levels [11]. Jaukovic et al. have shown that, at 200 °C, DON contents are reduced by 12% after 15 min and by 15% after 20 min of heating [16]. By contrast, another study reported more important reductions after frying flour artificially contaminated with DON. They reported reductions to 66%, 43%, and 38% observed at 169, 205, and 243 °C respectively [17,18]. It has to be noted that no toxicological study was conducted to ensure that molecules that may result from thermal decomposition of DON are safe and that, indeed, it can be considered as decontamination of food [15]. 

FUM are less thermoresistant than DON. Indeed, they have been reported to be stable up to only 100 °C [19] and recent experiments have shown that food processing may significantly reduce FUM levels. For example, decreases in FB1 were observed when frying and cooking contaminated corn-based foods at temperatures above 180 °C [20]. Dupuy et al. demonstrated that oven firing of maize samples at 150 °C for 40 min reduces the FB1 concentration by nearly 87% whereas lower temperatures and shorter exposure times resulted in little or no reduction [21]. Few data also suggest that FUM are also sensitive to microwave radiations and microwave cooking of popcorn maize significantly reduced FB1 concentration [22].

However, data on the effect of popcorn popping on FUM concentrations are quite limited and no studies have yet reported whether hot air and microwave popping methods influence the DON content.

Popcorn corn (*Zea mays var. everta*) is a type of horny starch corn with the particularity of containing almost exclusively hard starch and a hard pericarp in addition to a large quantity of water in the starch granules of the translucent endosperm. This allows it to “pop” [23]. Indeed, the popping phenomenon begins when the water in the pericarp reaches 100 °C, which leads to a thermodynamic equilibrium with water vapor, forming in essence a “small pressure cooker” [24]. At the critical point, the pericarp starts to pop, and the starch granules contained in the endosperm of the pericarp expand adiabatically to form a spongy complex. This phenomenon is called “expansion”. Numerous studies of the popping phenomenon in oven-heated popcorn have identified a critical popping temperature around 180 °C [24]. Proper popping requires a moisture content of around 13–14.5%, with the optimum being 13.5% [25,26].

The present study thus investigates the impact of various popcorn-popping techniques (hot air, hot oil, microwave) on DON and FUM contents on two types of popcorn maize samples.

## 2. Results and Discussion

### 2.1. FUM and DON Contents of the Popcorn Samples (Unpopped Controls)

All the samples in the study (*n* = 39) were analyzed by LC–MS/MS for FUM and DON. The levels of contamination observed in all these samples are reported in Table 1.

Concerning the FUM determinations, the results obtained show that 23% of the samples in the study were above the limit of 1000 µg/kg set by the European Union for direct human consumption [7,8]. Surveys carried out on maize contamination in Central Europe show average contaminations that are usually below 1000 µg/kg (average contaminations of positive samples measured in 2019 and in 2020 are 623 µg/kg and 552 µg/kg respectively) [27,28]. The levels of FUM contamination in this study are closer to those recorded in Eastern European maize production in recent years (average contamination close to the EU target of 1000 µg/kg with 1100 µg/kg in 2020) and to those recorded in the 2020 maize harvest across Europe (average of positive samples of 1153 µg/kg) [29]. In terms of prevalence, the incidence rate in the present study is also high compared to those recorded in Central Europe in 2019 and 2020 surveys (65% in 2019 and 42% in 2020) and is closer to the data obtained in 2020 for the whole Europe with 71% of contaminated samples [27,28,29]. Other studies report occurrences on maize samples collected across Europe and partially confirm the trends observed in this study [1,2]. A study carried out on samples collected between 2009 and 2011 in Central Europe shows averages comparable to those observed on the FUM in this study [1]. However, the occurrence is higher in this study than in the period 2009–2011 in Central Europe (60% reported) and is rather comparable to that reported on maize samples from Southern Europe (90% reported) [1] and to the worldwide study realized in maize samples collected across 100 countries from 2008 to 2017 by Gruber-Dorninger, Jenkins and Schatzmayr (80% of positive samples reported) [2]. For DON, 18% of the samples are above the EU limit of 750 µg/kg on products for direct human consumption [7,8]. The proportion of samples positive for this mycotoxin is lower than that of FUM. This proportion is comparable to that observed in the survey done in 2020 on the contamination of European maize samples (70% of positive samples reported) [29]. The average of the positive samples for this mycotoxin is comparable to that recorded by others in 2019 and 2020 (1026 and 808 µg/kg on average respectively) [28,29]. When the non-contaminated samples are considered, the overall average is lowered below the European limit, with 560 µg/kg. Considering the study carried out over the period 2009–2011, the positivity rate found in this study seems comparable to that found by Rodrigues and Naehrer in Central Europe (72% of positive samples reported) and by Gruber-Dorninger, Jenkins, and Schatzmayr (reduction of 67%) [1,2].

The samples used in this study are made of two different types of popcorn: Butterfly and Mushroom. These two types of maize differ mainly in their type of expansion and shape.

In order to evaluate the impact of popping on both FUM and DON content, three different methods were tested.

### 2.2. Impact of Popping Mode on FUM Contents

Microwave, air, and oil popping were tested and the FUM content of grains was analyzed before and after popping by LC–MS/MS.

Table 2 shows the difference in FUM content of unpopped (control) and popped popcorn samples (MW, AIR, and OIL) of the study (1% alpha risk).

The average FUM content of the control samples differs significantly from the contents of the popped samples (*n* = 156; *K_obs_* = 49.804; ddl = 3; *p*-value < 0.0001). Indeed, the average FUM content of the control samples (WP) is 1351 µg/kg (±352 µg/kg), decreasing to 192 µg/kg (±49 µg/kg) after microwave popping (−86%), 150 µg/kg (±61 µg/kg) after hot air popping (−89%), and 34.4 µg/kg (±17.5 µg/kg) after hot oil popping (−98%). The average FUM content decreases to 126 µg/kg (±36 µg/kg) when considering all popping methods (−91%). Hot oil popping reduces FUM content significantly more than the other two techniques (*n* = 156; *K_obs_* = 49.804; ddl = 3; *p*-value < 0.0001). The FUM contents for hot air and microwave popping do not differ significantly. The FUM content averaged over all popping methods differs significantly from that of the control sample (*n* = 156; *K_obs_* = 40.110; ddl = 1; *p*-value < 0.0001).

The reduction displayed here seems to follow the trends observed by Katta et al. and Vanara et al. in their respective studies on the distribution of FUM on maize grain [30,31]. Katta et al. observed the highest concentrations of FUM in the pericarp and germ, and ten times lower concentrations in the endosperm [30]. Vanara et al. showed that the pericarp of maize had the highest proportion of FUM (75–89%), the germ and endosperm having a low proportion of total grain FUM (6–9% and 6–8% respectively) [31]. The average decreases observed during this study seem to be close to the proportions contained in the pericarp, suggesting that the reduction in content may be due to the removal of the pericarp during popping. Since the spongy part of the popcorn resulting from the expansion consists almost exclusively of the endosperm containing the starch granules, the low amount of remaining toxins could correspond to the small proportion initially contained in that part of the grain.

Table 2 shows also the differences in FUM content before and after the popping of Butterfly and Mushroom popcorn for all three types of popping. Based on the standard deviations of the averages generated for these graphs, we cannot conclude that there is a difference in FUM contents between the two types of popcorn studied before and after popping. However, this data allows us to see that the reductions between Butterfly and Mushroom follow roughly the same trend. This study is the first one showing the effect of popping on the contents of FUM according to the type of popcorn and there is no data in the literature to locate this type of contamination on Butterfly and Mushroom popcorn.

From a statistic viewpoint, the average FUM content of the Butterfly unpopped (control) samples differs significantly from that of the AIR, OIL, and MW samples (*n* = 84; *K_obs_* = 19.206; ddl = 3; *p*-value = 0.0002) and, thus, from the average FUM content of all samples (ALL) (*n* = 84; *K_obs_* = 13.506; ddl = 1; *p*-value = 0.0002). As for the Butterfly popcorn, the average content of the Mushroom control samples differs significantly from that of the popped samples for all popping types (*n* = 72; *K_obs_* = 34.268; ddl = 3; *p*-value < 0.0001). Considering all popping modes combined reveals significant differences with the unpopped control samples (*n* = 72; *K_obs_* = 27.699; ddl = 1; *p*-value < 0.0001).

Then, Table 2 shows the content of FUM B1 (FB1) and FUM B2 (FB2) before and after popping. In this study, we can also see that there is a ratio close to 0.20 between FB1 and FB2 for unpopped controls and popped samples (MW, AIR, OIL, and ALL). Several studies described the occurrence of different types of FUM in the grain. Rheeder, Marasas, and Vismer reported that FB1 was the most abundant FUM in maize with proportions between 70–80%, FB2 being between 15 and 25% and FB3 between 3 and 8% of total FUM [32]. Another study reported a less proportion for FB1 in maize samples with 65.9%, but a similar proportion for FB2 [33].

We can see that the evolution of FB1 and FB2 content is comparable after popping. The same phenomenon had been observed during the processing of cornflakes [34,35]. These two data may explain the strong correlation between the FB1 and FB2 contents for samples popped in the same way, which is indicative of a similar evolution of these two molecules of FUM within the same sample despite the different contents (r^2^ = 0.925) that is close to the strong correlation of 0.95 observed by Zentai et al. between FB1 and FB2 in maize samples [36]. In addition, a significant difference is observed between the popped and unpopped (control) samples for these two FUM groups. Specifically, the content of the unpopped samples (controls) differs significantly from that of the popped samples for all three popping techniques for FB1 (*n* = 156; *K_obs_* = 49.404; ddl = 3; *p*-value < 0.0001) and FB2 (*n* = 156; *K_obs_* = 59.640; ddl = 3; *p*-value < 0.0001).

Significant differences appear also between the three popping techniques. The hot oil method proves to be the most effective method to eliminate FB1, whereas contents with the other two popping methods seem to evolve in a similar manner (*n* = 156; *Kobs* = 49.404; ddl = 3; *p*-value < 0.0001). Then, this study has shown that the content averaged over all popped samples differs from that of the unpopped controls for both FB1 (*n* = 156; *K_obs_* = 39.682; ddl = 1; *p*-value < 0.0001) and FB2 (*n* = 156; *K_obs_* = 55.074; ddl = 1; *p*-value < 0.0001).

The reduction in FUM concentration via popping appears to be greater and faster than that described in the existing literature, which may be due to the specificity of the popping method and to the type of popcorn used (case of expansion). This physical transformation of the grain may increase the destruction of mycotoxins attached to the matrix.

In order to better understand the differences obtained in this section, it is interesting to observe the distribution of the data as a whole.

**Figure 1:**
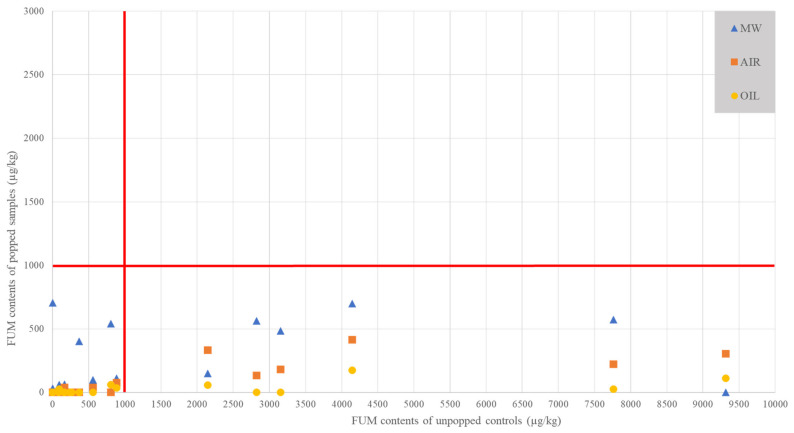
Comparison between the values obtained on the popped samples and the unpopped controls in FUM. Red lines correspond to the European limit of 1000 µg/kg for unpopped controls and popped samples. AIR= hot air popped samples; MW= microwave popped samples; OIL= oil popped samples.

Figure 1 shows the spatial comparisons between the values obtained for the unpopped controls and the values obtained with the three popping methods for all samples in the study. The purpose of this figure is to see if popping efficacy depends on the initial amount of FUM and to observe the distribution of samples with a value below the EU direct human consumption limit (1000 µg/kg) [7,8]. We do not look at the samples according to their percentage reduction but according to their range after popping (above or below the European limit). As observed in 2.1, we can observe ranges of contents from not detected to 9315 µg/kg on the unpopped controls. It can be noted here that almost all the samples analyzed fall below the limit of 1000 µg/kg regardless of the starting value. This trend shows that even if an increase in FUM may occur after popping, it will not be significant enough to raise the value above the European limit. Only two popped samples are affected by exceeding the limits in the figure. The first sample (MW), although showing an increase, cannot be considered to have had a very significant effect (very slight increase with a value of 1218 µg/kg for the unpopped control and 1326 µg/kg for the popped sample). The “AIR” sample, which is above the European limit, still shows a major reduction after popping (initial sample at 7046 µg/kg against 2295 µg/kg for the air popped sample). These two exceptional exceedances may be due to the initial sampling. Indeed, the complexity and heterogeneity of the contamination due to the matrix used could also be at the origin of these exceedances. Then, more widely, the trends observed in this figure demonstrate that, even with high initial contents, popping usually results in a reduction to a final content below the regulatory value. Another tendency to be observed on the FUM would therefore be the absence of a definite relationship between the extent of the reduction and the initial content of the non-popped controls.

### 2.3. Impact of Popping Mode on DON Contents

Three same types of popping (air, microwave, and oil) were also tested for their impact on DON content, analyzed before and after popping by LC–MS/MS.

Table 3 shows average DON content for unpopped and popped popcorn samples.

The average content of unpopped (control) samples is 560 µg/kg (+/−193 µg/kg). The content drops to 398 µg/kg (±124 µg/kg) upon hot air popping (−29%), to 284 µg/kg (±93 µg/kg) after microwave popping (−49%), and to 236 µg/kg (±88 µg/kg) after hot oil popping (−58%). The average DON content of popcorn popped by the three popping methods is 304 µg/kg (±94 µg/kg) (−46%). Although a clear trend to reduction is seen, no significant difference is detected in the DON content of the unpopped (control) samples versus the samples popped with hot air or microwaves. Hot oil popping, conversely, produces a significant reduction in DON content compared to the unpopped controls only for Mushroom. Finally, if we take a classic threshold of 5%, no significant difference in the DON content is apparent between the three popping methods (*n* = 156; *K*_obs_ = 6.916; ddl = 3; *p*-value = 0.075). Also, considering the average DON content in all popped samples reveals non-significant reduction compared to the content of unpopped (control) samples (*n* = 156; *K*_obs_ = 3.050; ddl = 1; *p*-value = 0.081). However, the *p*-values are close to the 5% threshold, which still gives us an interesting trend in the reduction even if it is not significant at 5% unless we tolerate a threshold at 10%. As for the FUM, the decrease in DON levels seems to follow the degradation of mycotoxins presents on the surface of the pericarp of the grain during popping (part removed during this process), but the proportion of DON present in this part of the grain appears to be lower than for FUM. Indeed, a study on the distribution of DON on maize grains showed that 55% of DON was in the pericarp [37]. This proportion is consistent with the reductions observed across all poppings and may explain the differences in reductions between DON and FUM in the study samples.

However, it can be seen that when the three techniques are analyzed separately, there are differences (particularly with hot air popping, where the overall reduction is lower and far from 55%). It would therefore be interesting to compare these data with those for unpopped grains during the popping process in order to see what impact the non-removal of the pericarp might have on these reductions in content.

The difference in reductions observed on DON compared to FUM can be explained in part by its greater thermoresistance, with instability observed between 170 and 350 °C, the popcorn popping being done at temperatures close to 200 °C [3]. Moreover, we can notice that the average percentages of reduction observed in this study seem to follow the same trends as for the other food processes, with the same variability [9].

Table 3 shows also the differences in average DON content of unpopped control samples and popped samples of Butterfly and Mushroom types.

For Butterfly samples, although the raw data indicate that the average contents decrease, the dispersion around the medians is too large to determine a significance between the different post-popping contents and the contents of the unpopped control samples (*n* = 84; *K_obs_* = 2.764; ddl = 3; *p*-value = 0.429) and between the average over all popped samples and the unpopped control samples (*n* = 84; *K_obs_* = 1.919; ddl = 1; *p*-value = 0.166).

For Mushroom type, no significant difference appears between the DON content of unpopped popcorn (WP) and the average DON content of popped Mushroom popcorn (ALL) (*n* = 72; *K_obs_* = 2.609; ddl = 1; *p*-value = 0.106). However, significant differences appear in the DON content between samples popped with hot oil (OIL) and those either popped with hot air (AIR) or unpopped (WP), whereas no significant difference appears between samples popped with hot oil and samples popped with microwaves (MW) (*n* = 72; *K_obs_* = 8.745; ddl = 3; *p*-value = 0.033).

Overall, popping Butterfly popcorn leads to a greater decrease in DON content than popping Mushroom popcorn. However, we can see throughout this study that the lower reductions on Mushroom are also related to a lower initial DON level.

In some cases, the absence of a significant difference in the DON content can be also explained by a strong disparity in the initial mycotoxin content. In fact, numerous popped samples had a higher DON content than the corresponding unpopped (control) samples, probably due to the complexity and heterogeneity of the matrix used, like for FUM: approximately 26% of the samples analyzed fall into this category (see Table A3), most of which are microwave-popped samples (20% of the samples analyzed). The same phenomenon also occurs for FUM, but only 12% of the samples are affected (cf. Table A2). Microwave popping again seems to be the popping method that provides the most deviations (≈10% of the samples concerned).

**Figure 2:**
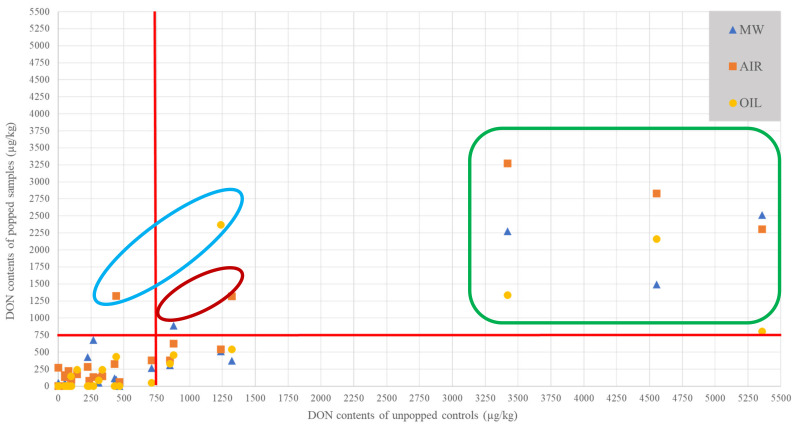
Comparison between the values obtained on the popped samples and the unpopped controls in DON. Red lines correspond to the European limit of 750 µg/kg for unpopped controls and popped samples. AIR= hot air popped samples; MW= microwave popped samples; OIL= oil popped samples.

As for the FUM previously, Figure 2 shows the spatial comparisons between the values obtained for the unpopped controls and the corresponding popped samples (MW, AIR, and OIL). The objective is to see the proportion of popped samples with a value below the EU limit for DON (750 µg/kg) taking into account the initial levels of their original unpopped controls [7,8].

Even if the trend is less obvious than for FUM (Table A1), it can be noted that most of the treated samples are below the 750 µg/kg set by the European authorities, especially for samples with low initial levels (below 1500 µg/kg). As with FUM, this trend shows that an increase in levels after popping does not necessarily mean that these limits are exceeded (Table A2). There are, however, samples where an increase was observed after popping and which are above the EU limit of 750 µg/kg (blue box). The samples in the red box, although above the 750 µg/kg limit, do not show an increase after popping but rather correspond to an absence of variation with the unpopped control. Finally, it can also be noted that the samples with high initial levels (>3000 µg/kg) do not give values below the limits set by the European authorities despite a notable and significant decrease for most of them (green box).

This phenomenon may be explained in part by the hypothesis of mycotoxin stability during food processing. Previous studies have shown that DON is present in the raw material with its associated masked or conjugated forms, such as DON-3-Glucoside (DON-3-Glc), 15-Acetyl-DON (15ADON), and 3-Acetyl-DON (3ADON). Kostelanska et al. reported that the ratios are in the range 0.07–0.29 for DON-3-Glc with DON on various cereals such as corn or wheat [38]. The likely ability of these modified forms to transform into native forms could, for example, lead to increased levels of DON during food storage or food processing. The latter, being less heat stable, converts to the native form during food processing, as occurs, for example, in the baking process of bakery products [39]. At the same time, Vidal et al. assert that some of the toxins may be incorporated into the plant matrix and therefore are not measurable in the analysis [39]. The toxins can be made quantifiable during physical transformation processes such as cooking or fermentation. Mycotoxin-extraction methods must therefore be as exhaustive as possible to quantify the proportion of these non-quantifiable mycotoxins that are strongly linked to the matrix [40]. Additional assays of modified forms and strongly matrix-related mycotoxins would be helpful to explain these phenomena and refine these conclusions.

## 3. Conclusions

Popcorn has the particularity of popping at a high temperature. Although different food processing methods and parameters such as temperature have been tested on their ability to reduce mycotoxin content, no studies have focused heretofore on how popping affects these contaminants.

Thus, the present work focuses on how three popcorn-popping methods (hot air, hot oil, and microwave) affect the DON and FUM contents of the popcorn. For all samples measured, the results reveal reductions in DON and FUM contents upon popping.

However, the magnitude of the reduction depends on the mycotoxin. Popping causes a more substantial and statistically significant reduction for FUM than for DON. Moreover, FUM is more thermosensitive and is located in a high proportion in the pericarp that will be removed during the popping process which can explain the higher reductions for this mycotoxin.

The reduction in DON or FUM content upon popping also depends on the popping techniques, with hot oil popping leading to the greatest reduction in DON and FUM contents. Conversely, hot air and microwave popping lead to roughly the same reduction in DON and FUM contents. These differences may be due to temperature as well as the uncontrollable and random nature of popcorn popping.

More widely, this study demonstrates that the popping process is a good way to reduce exposure to these toxins often present on the grain.

## 4. Materials and Methods

### 4.1. Popcorn Samples

Thirty-nine samples of popcorn weighing approximately 1 kg each of naturally contaminated popcorn were collected in 2019 in southwest France. Sampling was stratified to include both types of popcorn (Butterfly and Mushroom) in nearly equal proportions (21 samples for Butterfly and 18 samples for Mushroom) and a range of levels for each type. These types differ in their popping performance and in the type of expansion upon popping. Butterfly type generally undergoes a larger volumetric expansion than Mushroom type, which has a coarser shell that often adheres to the popped grain [41].

Samples were stored in a controlled atmosphere and allowed to dry until reaching a moisture content appropriate for optimal popping [25,26].

For assays, each sample was homogenized and divided for assays into four subsamples identical to the rifle divider (Haver & Boeker, Oelde Germany). The mass of subsamples (*n*= 156) was adapted to each popping technique (Table 4).

### 4.2. Popping

#### 4.2.1. Popping with Hot Air (AIR)

Popping was done by using a Cretors^®^ hot air popper (C. Cretors & Co., Model MWVT DIGITAL 9575E, Chicago, IL, USA). 0.25 kg of corn was heated to 215 °C (419 °F) for 5 min. No prior handling and addition (e.g., oil) was required for this technique.

#### 4.2.2. Popping with Hot Oil (OIL)

Popping was done in a Cretors^®^ oil popper (C. Cretors & Co., Model FT-80P-E-M, Chicago, IL, USA): 0.115 kg of palm oil were added to 0.25 kg of corn, and the whole was heated to 249 °C (480 °F) for 5 min.

#### 4.2.3. Popping with Microwave (MW)

For this technique, 0.084 kg of popcorn corn and 0.016 kg of palm oil were placed in a standard bag (Weaver, Van Buren, IN, USA) for microwave popping. Each bag was then popped in a microwave oven for 3 min at 1000 W (Samsung., Model MS28J5215AW, Tokyo, Japan). The temperature inside the grain reached 200 °C (392 °F) at popping.

### 4.3. Determination of FUM and DON Contents

DON and FUM (B1 + B2) were determined in unpopped grains (WP) as control samples and in popped grains for the AIR, OIL, and MW samples. Mycotoxin content was determined by a private accredited institution (Phytocontrol^®^, Nimes, France—http://www.phytocontrol.com/), using accredited methods. This institution is specialized in pesticide and contaminant residue detection with accreditation ISO 17025 delivered by French Council for Accreditation, Audit, and Control (COFRAC) [42]. DON and FB1 and FB2 mycotoxins were extracted using the method described and validated by Lehotay et al. [43]. The linearity, recovery, repeatability, limit of detection, and limit of quantification of the method were >0.99, 83% for DON—72% for FB1 and 78% for FB2, 23% for DON—30% for FB1 and 28% for FB2, 50 µg/kg for DON and 25 μg/kg for FUM, and 25 µg/kg for DON and 12.5 μg/kg for FUM, respectively. These recovery percentages do not vary, whether the grains were unpopped or popped, and whatever the popping method used. The LODs and LOQs were defined as the concentrations that gave a signal to noise ratio (S/N) of 3:1 and 10:1, respectively. Before mycotoxin determination, each of the 156 sub-samples was ground entirely (RETSCH., Model 19260 GM 300, Germany) at 4000 rpm for 3 min. After grinding, 5 g of sample were mixed with 20 mL of demineralized water and acetonitrile (50:50). After 10 min of agitation, a mixture of anhydrous magnesium sulfate (4 g), sodium chloride (1 g), trisodium citrate dihydrate buffer (1 g), and disodium hydrogenocitrate sesquihydrate (0.5 g) (Sigma-Aldrich, St. Quentin-Fallavier, France) salts (pH 5 to 5.5) is added. This mixture is then shaken vigorously, and the sample is centrifuged to separate the phases. The final extract is diluted after evaporation and analyzed by LC–MS/MS (Shimadzu, LC-MS model 8060, Kyoto, Japan) using an ESI+ interface for FUM and ESI- for DON was used. The mobile phases were solvent A (1 mM ammonium formate and 0.5% acetic acid in water) and solvent B (1 mM ammonium formate and 0.5% acetic acid in methanol). The elution was carried out using a specific gradient from 0 to 4 min (Table 5).

The injection volume set was 20 µL, the nebulizer, the heating gas, and drying gas were set at 3, 10, and 10 L.min^−1^ respectively. The interface, desolvation line, and heat block temperature were 300 °C, 250 °C, and 400 °C, respectively. The ions transitions used for the mycotoxins identification and quantification are represented in Table 6.

The concentrations used for the standards curves of FUM and DON used for the quantification of mycotoxins are presented in Table 7.

### 4.4. Statistical Analysis

The statistical analyses were done using Xlstat software (version 2019.3.2; Addinsoft, XLSTAT statistical and data analysis solution, Long Island, NY, USA; https://www.xlstat.com 2019). To study how the different popping modes affect the DON and FUM contents, we compared the variance of all data obtained by using a non-parametric Kruskal–Wallis test at 1%, 5%, and 10% and a Dunn test.

## Figures and Tables

**Table 1:** FUM and DON contents of the 39 unpopped popcorn samples (controls) of the study. For this study, two types of popcorn (Butterfly (*n* = 21) and Mushroom (*n* = 18)) were analyzed by LC–MS/MS. The limits of quantification (LOQ) for both FUM and DON were 25 µg/kg and 50 µg/kg, respectively. The limits of detection (LOD) for both FUM and DON were 12.5 µg/kg and 25 µg/kg, respectively. For the purpose of the study, values below the LOD were set to zero, values below the LOQ were reported as the LOQ (25 µg/kg for FUM and 50 µg/kg for DON). A sample is considered positive when its mycotoxin content is above the LOQ.

Popcorn Types		FUM (FB1 + FB2)	DON
All samples (*n* = 39)	Samples < LOD	5 (13%)	12 (30%)
	Samples > EU regulation	9 (23%)	7 (18%)
	Average (µg/kg)	1351	560
	Average of positive samples (µg/kg)	1550	809
	Maximum (µg/kg)	9315	5359
Butterfly (*n* = 21)	Samples < LOD	5 (24%)	4 (19%)
	Samples > EU regulation	6 (29%)	6 (29%)
	Average (µg/kg)	1570	943
	Average of positive samples (µg/kg)	2061	1165
	Maximum (µg/kg)	9315	5359
Mushroom (*n* = 18)	Samples < LOD	0 (0%)	8 (44%)
	Samples > EU regulation	3 (17%)	1 (6%)
	Average (µg/kg)	1097	113
	Average of positive samples (µg/kg)	1097	204
	Maximum (µg/kg)	7046	878

**Table 2:** Decrease in average and median FUM (FB1 + FB2), FB1 and FB2 contents for the three popping methods in 39 maize samples (Butterfly and Mushroom)—alpha risk 1%. Each of the 39 starting samples was divided into 4 sub-samples for a total of 156 sub-samples analyzed in FUM by LC–MS/MS: 39 unpopped control sub-samples (WP)/39 microwave sub-samples popped at 200 °C with 0.016 kg of palm oil for 3 min at 1000W (MW)/39 hot air sub-samples popped at 215 °C (AIR)/39 hot oil sub-samples popped at 249 °C with 0.115 kg of palm oil (OIL). To investigate the reduction percentages for each technique, the average was calculated for each group of sub-samples and non-parametric tests of Kruskal–Wallis and Dunn (statistic groups) have been made to compare them. /ALL = (AIR + OIL + MW)/*** = *p*-value < 0.001/^a^, ^b^, … = Dunn test groups.

	Popping Method	FUM Average (µg/kg)	FUM Average Reduction	FUM Median (µg/kg)	FUM Median Reduction	FUM Interquartile Range (µg/kg)
All samples	WP	1351		552 ^b^ ***		830
	MW	192	−86%	54 ^a^ ***	−90%	213
	AIR	150	−89%	37 ^a^ ***	−94%	181
	OIL	34	−98%	0 ^a^ ***	−100%	25
	ALL	126	−91%	25 ^a^ ***	−96%	100
Butterfly	WP	1570		264 ^b^ ***		2059
	MW	213	−86%	63 ^ab^ ***	−76%	483
	AIR	83	−95%	0 ^a^ ***	−100%	133
	OIL	24	−99%	0 ^a^ ***	−100%	25
	ALL	107	−93%	0 ^a^ ***	−100%	112
Mushroom	WP	1097		765 ^b^ ***		573
	MW	167	−85%	39 ^a^ ***	−95%	96
	AIR	229	−79%	47 ^a^ ***	−94%	164
	OIL	47	−96%	0 ^a^ ***	−100%	19
	ALL	148	−87%	25 ^a^ ***	−97%	69
	**Popping Method**	**FB1 Average (µg/kg)**	**FB1 Average** **Reduction**	**FB1 Median (µg/kg)**	**FB1 Median** **Reduction**	**FB1** **Interquartile Range**
All samples	WP	1104		466 ^b^ ***		655
	MW	159	−86%	54 ^a^ ***	−88%	213
	AIR	125	−89%	37 ^a^ ***	−92%	181
	OIL	32	−97%	0 ^a^ ***	−100%	25
	ALL	105	−91%	25 ^a^ ***	−95%	100
Butterfly	WP	1277		200 ^b^ ***		1541
	MW	182	−86%	63 ^ab^ ***	−69%	399
	AIR	78	−94%	0 ^ab^ ***	−100%	133
	OIL	24	−98%	0 ^a^ ***	−100%	25
	ALL	95	−93%	0 ^a^ ***	−100%	112
Mushroom	WP	901		612 ^b^ ***		453
	MW	133	−85%	39 ^a^ ***	−94%	96
	AIR	180	−80%	47 ^a^ ***	−92%	180
	OIL	41	−95%	0 ^a^ ***	−100%	19
	ALL	118	−87%	25 ^a^ ***	−96%	69
	**Popping Method**	**FB2 Average (µg/kg)**	**FB2 Reduction**	**FB2 Median (µg/kg)**	**FB2 Median** **Reduction**	**FB2** **Interquartile Range**
All samples	WP	253		98 ^b^ ***		182
	MW	33	−87%	0 ^a^ ***	−100%	15
	AIR	28	−89%	0 ^a^ ***	−100%	0
	OIL	3	−99%	0 ^a^ ***	−100%	0
	ALL	21	−92%	0 ^a^ ***	−100%	0
Butterfly	WP	296		64 ^b^ ***		330
	MW	32	−89%	0 ^ab^ ***	−100%	63
	AIR	8	−97%	0 ^a^ ***	−100%	0
	OIL	0	−100%	0 ^a^ ***	−100%	0
	ALL	13	−96%	0 ^a^ ***	−100%	0
Mushroom	WP	202		121 ^b^ ***		111
	MW	34	−83%	0 ^a^ ***	−100%	0
	AIR	52	−74%	0 ^a^ ***	−100%	0
	OIL	6	−97%	0 ^a^ ***	−100%	0
	ALL	31	−85%	0 ^a^ ***	−100%	0

**Table 3:** Decrease in average and median DON contents for the three popping methods in all maize samples (Butterfly and Mushroom)—alpha risk 5%. Each of the 39 starting samples was divided into 4 sub-samples for a total of 156 sub-samples analyzed in FUM by LC–MS/MS: 39 unpopped control sub-samples (WP)/39 microwave sub-samples popped at 200 °C with 0.016 kg of palm oil for 3 min at 1000W (MW)/39 hot air sub-samples popped at 215 °C (AIR)/39 hot oil sub-samples popped at 249 °C with 0.115 kg of palm oil (OIL). To investigate the reduction percentages for each technique, the average was calculated for each group of sub-samples and non-parametric tests of Kruskal–Wallis and Dunn (statistic groups) have been made to compare them. /ALL = (AIR + OIL + MW)/** = *p*-value from 0.001 to 0.01; * = *p*-value from 0.01 to 0.05/^a^, ^b^, … = Dunn test groups.

	Popping Method	DON Average (µg/kg)	DON Average Reduction	DON Median (µg/kg)	DON Median Reduction	DON Interquartile Range
All samples	WP	560		80 ^b^ *		436
	MW	284	−49%	50 ^ab^ *	−38%	248
	AIR	398	−29%	98 ^ab^ *	+23%	305
	OIL	236	−58%	0 ^a^ *	−100%	192
	ALL	304	−46%	0 ^a^ *	−100%	266
Butterfly	WP	943		309 ^a^ *		779
	MW	432	−54%	117 ^a^ *	−62%	325
	AIR	646	−31%	174 ^a^ *	−44%	541
	OIL	416	−56%	89 ^a^ *	−71%	430
	ALL	498	−47%	143 ^a^ *	−54%	428
Mushroom	WP	113		50 ^b^ *		73
	MW	112	−1%	0 ^ab^ **	−100%	70
	AIR	95	−16%	0 ^b^ *	−100%	133
	OIL	25	−78%	0 ^a^ *	−100%	0
	ALL	78	−32%	0 ^b^ *	−100%	59

**Table 4:** Table of sample masses for each group of subsamples.

Nature of Sample	Mass (kg)
Microwave popping (MW)	0.084
Oil popping (OIL)	0.25
Air popping (AIR)	0.25
Without popping (WP)	0.25

**Table 5:** Gradient program of the LC–MS/MS system.

Time	Solvent A (%)	Solvent B (%)
0.01	90	10
1.50	45	55
3.50	15	85
4.00	15	85
4.01	98	2

**Table 6:** Ions Multiple Reaction Monitoring (MRM) and transitions.

Mycotoxin	*m*/*z*	Transition
DON	355 →95.1	Quantifiying
DON	355 → 59	Confirmation 1
DON	355 →265.1	Confirmation 2
FB1	722.35 → 334.4	Quantifiying
FB1	722.35 → 352.4	Confirmation 1
FB1	722.25 → 141.3	Confirmation 2
FB2	706.2 → 336.4	Quantifiying
FB2	706.2 → 318.4	Confirmation 1

**Table 7:** Standard concentrations used for the quantification of DON and FUM (FB1+FB2).

	Concentration (µg/L)				
	Std 1	Std 2	Std 3	Std 4	Std 5
DON	5	50	100	200	500
FB1-FB2	2.5	25	50	100	250

## Data Availability

The data presented in this study are available on request from the corresponding author.

## Appendix A

Table A1, Table A2 and Table A3 summarize the data.

**Table A1:** Summary of significant differences between the tested modalities.

	FUM				DON			
	MW vs. WP	AIR vs. WP	OIL vs. WP	ALL vs. WP	MW vs. WP	AIR vs. WP	OIL vs. WP	ALL vs. WP
Butterfly	Yes ***	Yes ***	Yes ***	Yes ***	No	No	No	No
Mushroom	Yes ***	Yes ***	Yes ***	Yes ***	No	No	Yes *	No
Butterfly and Mushroom	Yes ***	Yes ***	Yes ***	Yes ***	No	No	No	No
	**MW vs AIR**	**OIL vs AIR**	**OIL vs MW**		**MW vs AIR**	**OIL vs AIR**	**OIL vs MW**	
Butterfly	No	Yes **	Yes**		No	No	No	
Mushroom	No	Yes **	Yes **		No	Yes *	No	
Butterfly and Mushroom	No	Yes **	Yes **		No	No	No	

*** = *p*-value < 0.001; ** = *p*-value from 0.001 to 0.01; * = *p*-value from 0.01 to 0.05.

**Table A2:** The proportion of popped samples with greater FUM content than in the unpopped control samples.

	Butterfly		Mushroom		Butterfly and Mushroom	
Type of Popping	Proportion > WP	Number > WP	Proportion > WP	Number > WP	Proportion > WP	Number > WP
AIR	0%	0	6%	1	3%	1
OIL	0%	0	0%	0	0%	0
MW	14%	3	6%	1	10%	4
Total samples with at least one popped > WP	14%	3	11%	2	13%	5
Total samples	21		18		39	

**Table A3:** The proportion of popped samples with greater DON contents than in the unpopped control samples.

	Butterfly		Mushroom		Butterfly and Mushroom	
Type of Popping	Proportion > WP	Number > WP	Proportion > WP	Number > WP	Proportion > WP	Number > WP
AIR	10%	2	17%	3	13%	5
OIL	14%	3	0%	0	8%	3
MW	14%	3	28%	5	21%	8
Total samples with at least one popped > WP	19%	4	33%	6	26%	10
Total number of samples	21		18		39	
